# Y-Chromosome and mtDNA Genetics Reveal Significant Contrasts in Affinities of Modern Middle Eastern Populations with European and African Populations

**DOI:** 10.1371/journal.pone.0054616

**Published:** 2013-01-30

**Authors:** Danielle A. Badro, Bouchra Douaihy, Marc Haber, Sonia C. Youhanna, Angélique Salloum, Michella Ghassibe-Sabbagh, Brian Johnsrud, Georges Khazen, Elizabeth Matisoo-Smith, David F. Soria-Hernanz, R. Spencer Wells, Chris Tyler-Smith, Daniel E. Platt, Pierre A. Zalloua, The Genographic Consortium

**Affiliations:** 1 The Lebanese American University, Chouran, Beirut, Lebanon; 2 Institut de Biologia Evolutiva (CSIC-UPF), Departament de Ciències de la Salut i de la Vida, Universitat Pompeu Fabra, Barcelona, Spain; 3 Modern Thought and Literature, Stanford University, Stanford, California, United States of America; 4 Allan Wilson Centre for Molecular Ecology and Evolution, University of Otago, Dunedin, New Zealand; 5 The Genographic Project, National Geographic Society, Washington, DC, United States of America; 6 The Wellcome Trust Sanger Institute, Wellcome Trust Genome Campus, Hinxton, United Kingdom; 7 Computational Biology Centre, IBM TJ Watson Research Centre, Yorktown Heights, New York, United States of America; 8 Harvard School of Public Health, Boston, Massachusetts, United States of America; University of Florence, Italy

## Abstract

The Middle East was a funnel of human expansion out of Africa, a staging area for the Neolithic Agricultural Revolution, and the home to some of the earliest world empires. Post LGM expansions into the region and subsequent population movements created a striking genetic mosaic with distinct sex-based genetic differentiation. While prior studies have examined the mtDNA and Y-chromosome contrast in focal populations in the Middle East, none have undertaken a broad-spectrum survey including North and sub-Saharan Africa, Europe, and Middle Eastern populations. In this study 5,174 mtDNA and 4,658 Y-chromosome samples were investigated using PCA, MDS, mean-linkage clustering, AMOVA, and Fisher exact tests of *F_ST_*'s, *R_ST_*'s, and haplogroup frequencies. Geographic differentiation in affinities of Middle Eastern populations with Africa and Europe showed distinct contrasts between mtDNA and Y-chromosome data. Specifically, Lebanon's mtDNA shows a very strong association to Europe, while Yemen shows very strong affinity with Egypt and North and East Africa. Previous Y-chromosome results showed a Levantine coastal-inland contrast marked by J1 and J2, and a very strong North African component was evident throughout the Middle East. Neither of these patterns were observed in the mtDNA. While J2 has penetrated into Europe, the pattern of Y-chromosome diversity in Lebanon does not show the widespread affinities with Europe indicated by the mtDNA data. Lastly, while each population shows evidence of connections with expansions that now define the Middle East, Africa, and Europe, many of the populations in the Middle East show distinctive mtDNA and Y-haplogroup characteristics that indicate long standing settlement with relatively little impact from and movement into other populations.

## Introduction

As a crossroad between Africa, Arabia, Asia, and Europe, the Levant has been a primary historical stepping stone in the first modern human expansions out of Africa and for later migrations into and out of Europe, Asia, and Africa [1]–[7]. As such, it has also become a land of remarkable human diversity. The earliest fossil and archaeological evidence of modern humans outside of the African continent are from the Levant, presumably indicating a migration via the northern route, and date to 125–95 kya [8], [9]. Additionally, genetic studies suggest that the initial peopling of Eurasia occurred through the northern Levantine (modern day Lebanon and Syria) route [10]–[12]. Two proposed routes chart the dispersal of anatomically modern humans out of the African continent: (1) a northern route, reaching west and central Asia through the Sinai Peninsula and the Levant, and (2) a southern route via the Bab el-Mandeb Strait and along the south Asian coast, ultimately reaching Australia [13]–[15].

While the out-of-Africa migrations have been major determining factors, other migratory events have strongly influenced genetic marker distributions throughout the Levant and the surrounding geographical areas. During the last glacial maximum (LGM, 26.5–19 kya), most of the Levant was an uninhabitable desert, with forested hills in Levantine Mediterranean coastal areas [7]. The genetics of the modern Levant were largely determined by subsequent repopulation (especially during the Neolithic agricultural revolution) and mass movements associated with empire building. Neolithic expansions in particular, beginning around 10 kya, induced gene flow between the Fertile Crescent and Europe, which shaped the genetic structure of both regions [16]–[22].

Most genetic studies of the Levant as a geographical area have focused exclusively on either Y-chromosome [23]–[27] or mitochondrial markers [28]. Further, contrasts between Y-chromosome and mtDNA data provide distinct insights into human expansions unavailable to somatic genome analyses [29]. While comparative analyses among the two marker types have been undertaken in the Middle East and Africa [30]–[34], none of these studies have explored the contrasting relationships of expansions throughout Europe, North Africa, the Levant and Arabian Peninsula after the LGM. Building on a previous study that reported phylogeographic characteristics of Y-chromosome markers in the Levantine region [25], we now compare and contrast Y and mtDNA phylogeographic distributions in the Levant and investigate the affinities of Middle Eastern populations with European and African populations.

## Materials and Methods

### Ethics statement

The samples were collected from donors after they had given their written informed consent to the project and to the data analysis, which was approved by the IRB of the Lebanese American University.

### mtDNA data

3,663 mtDNA records collected from the literature represented populations from Burkina-Faso [35], Cyprus [36], Egypt [37], Ethiopia [38], France [39], [40], Greece [36], Iraq [31], Jordan [30], Kenya [41], Libyan Sahara [42], Mali [35], Morocco [43], [44], Niger [35], Saudi Arabia [45], Slovakia [46], Tunisia [44], [47], and Yemen [38], [48]. In addition to this data, we added 1,511 new samples from Lebanon, Libya, Jordan, Palestine, and Syria. Samples were collected from unrelated blood donors from five countries. Surname repetitions were avoided and used as a criterion for absence of relatedness among volunteers, appropriate for Y-chromosome analysis. All demographic data were provided by self-assignment.

**Figure 1:**
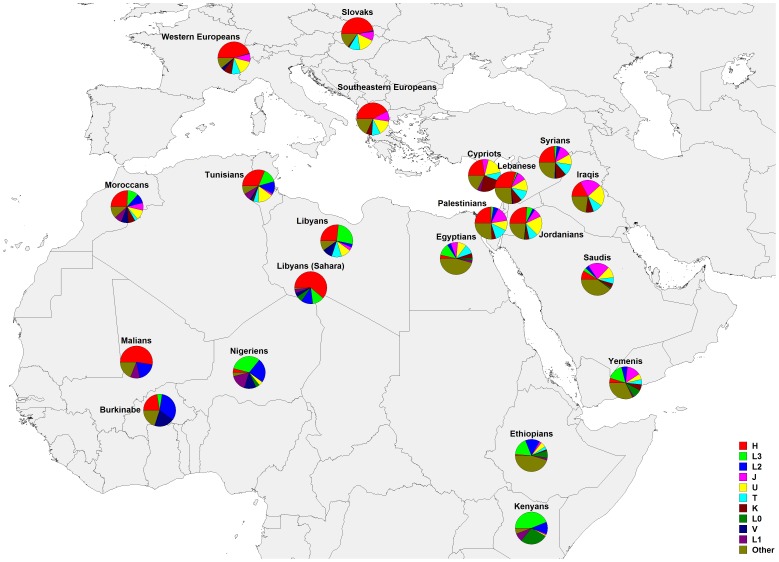
Geographic distribution of mtDNA haplogroups. Frequencies distribution from the current study and from the published data [30], [31], [35]–[48] as reported in Table 1.

Given the broad cultural and genetic diversity in the region, terms such as “Middle East” may be problematical. Historically, the term evolved during the era of European Imperialism, and included all lands between Arabia and India, but came to include Turkey through Saudi Arabia, extending east through Afghanistan and Pakistan. In this report, “Middle Easterners” refers to Iraqis, Jordanians, Lebanese, Palestinians, Saudis, Syrians, and Yemenis. The Greek data represent the Southeastern Europe region for mtDNA analyses, and is labelled “Southeastern Europe” in the rest of this report. France represents Western Europe mtDNA, and is labelled “Western Europe” throughout the rest of this report. These are reported in Table S1. All haplogroups were reduced to the most informative sets for the purpose of homogeneous representation and comparative analyses. mtDNA haplogroup frequencies are displayed in Table 1 and shown as pie charts in Figure 1.

**Table 1:** mtDNA Haplogroup frequencies of 1509 newly sequenced Levantine samples and 3665 samples collected from the literature.

Country	n	L*	L3*	M*	D	N*	R*	R0	HV	H	U	References
**Levant**												
Lebanon	979	0.6	1.4	1.4	0.2	10.5	21.2	5.0	8.5	29.9	21.1	This study
Syria	234	3.4	2.6	0.9	0.0	11.1	25.6	2.6	9.0	24.4	20.5	This study
Palestine	120	5.0	2.5	3.3	0.0	8.3	27.5	5.8	8.3	25.0	14.2	This study
Cyprus	79	3.8	0.0	2.5	0.0	12.7	17.7	0.0	0.0	22.8	40.5	Irwin et al. [36]
**Europe**												
France	871	0.6	0.3	0.1	0.0	5.4	18.0	0.5	6.9	45.4	22.8	Dubut et al. [39]Richard et al. [40]
Greece	372	0.0	0.0	1.1	0.0	9.1	19.4	1.9	5.9	42.2	20.4	Irwin et al. [36]
Slovakia	200	1.0	0.0	1.0	0.0	10.0	20.0	0.0	4.0	47.0	17.0	Malyarchuk et al. [46]
**Arabian Peninsula**												
Jordan	290	4.1	5.9	1.4	0.3	10.7	17.9	3.4	6.9	26.2	23.1	This studyGonzales et al. [30]
Iraq	52	0.0	0.0	1.9	0.0	0.0	34.6	5.8	11.5	17.3	28.8	Al-Zahery et al. [31]
Saudi Arabia	539	6.5	3.5	7.1	0.0	12.2	28.0	18.2	0.7	8.7	15.0	Abu-Amero et al. [45]
Yemen	300	22.0	16.3	5.3	0.0	6.7	21.7	9.3	2.3	4.7	11.7	Kivisild et al. [38]Cerny et al. [48]
**North Africa**												
Egypt	278	8.3	12.2	6.8	0.0	10.4	21.6	17.27	6.1	0.0	13.7	Saunier et al. [37]
Libya	31	6.5	25.8	0.0	0.0	3.2	12.9	6.5	9.7	25.8	9.7	This study
Libyan Sahara	129	21.7	11.6	1.6	0.0	0.0	0.0	0.0	3.9	61.2	0.0	Ottoni et al. [42]
Morocco	137	18.2	10.9	7.3	0.0	2.2	10.2	3.6	7.3	23.4	16.8	Turchi et al. [44]Harich et al. [43]
Tunisia	160	20.6	14.4	1.9	0.0	0.6	7.5	0.0	7.5	31.3	16.3	Cherni et al. [47]Turchi et al. [44]
**East sub Saharan**												
Ethiopia	232	36.6	18.1	14.7	0.0	3.9	5.2	11.6	2.6	0.9	6.5	Kivisild et al. [38]
Kenya	84	47.6	44.0	4.8	0.0	0.0	1.2	1.2	0.0	0.0	1.2	Brandstätter et al. [41]
**West sub Saharan**												
Burkina-Faso	40	35.0	5.0	17.5	0.0	0.0	0.0	0.0	20.0	22.5	0.0	Pereira et al. [35]
Mali	21	28.6	0.0	19.0	0.0	0.0	0.0	0.0	0.0	52.4	0.0	Pereira et al. [35]
Niger	25	44.0	32.0	4.0	0.0	0.0	0.0	0.0	12.0	4.0	4.0	Pereira et al. [35]

### Y-Chromosome data

1,774 previously published Y-chromosome records were obtained from the literature representing populations from the Balkans [49], Burkina-Faso [50], Ethiopia [50], Italy [51], Kenya [50], Saudi Arabia [52], Slovakia [53], and Yemen [52]. In addition 2,884 previously published data from our laboratory representing populations from Cyprus, Egypt, Lebanon, Libya, Jordan, Morocco, Palestine, Syria and Tunisia were added to this study. The Italian Y-chromosome samples represent Western Europe in this study, and are labelled “Western European” through the rest of this report. The Balkan samples represent the Southeastern Europe region, and are labelled “Southeastern European” in the rest of this report. These are reported in Table S2, with haplogroup frequencies reported in Table S3. The geographical haplogroup frequency distributions are displayed in Figure S1. Haplogroups of the Saudi, Yemeni, and Slovak populations were not available, thus we have predicted those haplogroups using the populations haplotypes and the online haplogroup prediction tool [54], [55]: www.hprg.com/hapest5/hapest5a/hapest5.htm. We have also computed STR-predicted Y haplogroups across populations that had been SNP defined to ascertain STR-based haplogroup assignment accuracy, and identify geographically correlated trends in assignment error rates that may impact our conclusions.

### mtDNA sequencing and in silico prediction of haplogroups

Total DNA was extracted from the peripheral leukocyte fraction of whole blood drawn in EDTA anticoagulant or cheek swab samples using a standard phenol/chloroform extraction procedure. The hypervariable region I (HVS-I) was amplified using primers designed by Maca-Meyer et al. [12]. Amplified HVS-I products were sequenced using a forward primer at position 15876 and a reverse primer at position 639 with ABI Big Dye Terminator v3.1 Cycle Sequencing kit (Applied Biosystems) and analysed on an Applied Biosystems 3130 xl Genetic Analyser.

Mutations in the HVS-I region were defined by aligning and comparing the sequences to the revised Cambridge Reference Sequence (rCRS) using the SeqScape software.

mtDNA haplogroups were predicted using the Genographic Project's online haplogroup prediction tool: nnhgtool.nationalgeographic.com.

### mtDNA Genotyping of samples

Haplogroup affiliations were confirmed using the Taqman approach with customized primers and probe sets to identify the SNPs listed in Table S4 (Applied Biosystems). Samples with incompatible prediction and Taqman results were excluded from the study. Mitochondrial nomenclature was assigned according to prior studies since established as standards [38], [56]–[59]. Data archiving was manually organized and edited.

### Reduction to most informative derived set of Haplogroups

MtDNA Haplogroups reported in the literature were updated and reconciled to the 2009 phylogeny reported by van Oven et al. [59]. Construction of the most informative derived set was achieved by identifying the maximum level of resolution shared across all included studies. If some subhaplogroup markers were not typed in any given study, but no samples in that study resolved to a less-derived paragroup, then the most derived resolution was retained for the constructed most informative derived set.

Further, HVS-I regions reported by different sources varied. The range representing the largest common subset of HVS-I SNPs reported included 16090 through 16365. Identified SNPs are reported in Table S5.

### Statistical analyses

#### Fisher Exact Tests

Fisher exact tests were performed for haplogroup frequencies within populations. These tests were performed against a background of all populations (Table S6), as well as among Middle Eastern populations (from Iraq, Jordan, Lebanon, Palestine, Saudi Arabia, Syria, and Yemen) only (Table S7), with very low-power tests excluded.

#### PCA

**Figure 2:**
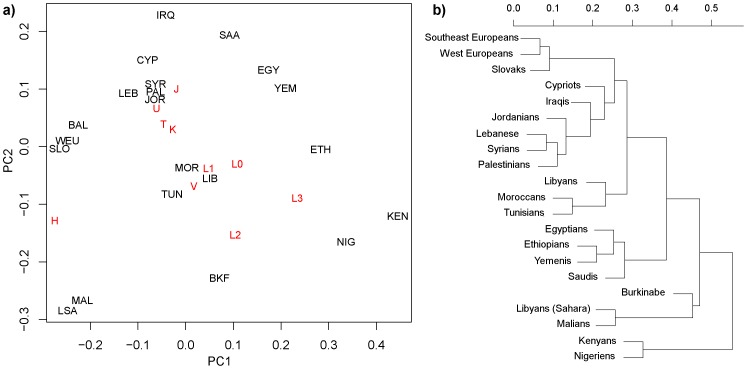
Populations comparison based on mtDNA haplogroups. a) Principal Component Analysis of relative frequencies of haplogroups within populations, b) with mean-linkage (UPGMA) dendrogram determined from Euclidean distances.

Numbers of samples bearing mtDNA and Y-Chromosome reduced haplogroups within each population, and relative haplogroup frequencies within populations, were computed using R [60]. Principal Component Analysis was computed using prcomp in R [61]. Results were displayed with principal component contributions from each haplogroup using biplot. Agglomerative clustering with mean linkage (UPGMA) was applied to Euclidean distances computed between relative frequency vectors for each population using agnes and displayed in Figure 2 for mtDNA Haplogroups and Figure S2 for Y-Haplogroups. These dendrograms should not be taken as population histories, but rather provide a repeatable description of population similarities also visible in the PCA.

#### MDS

**Figure 3:**
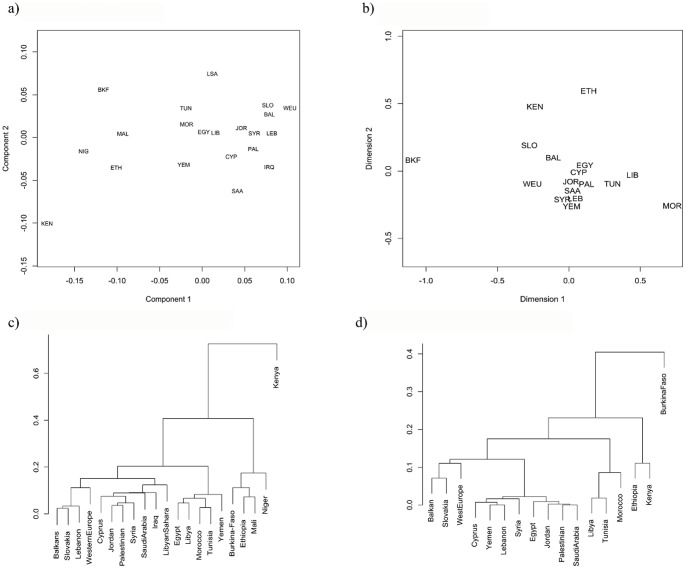
Nonmetric Multidimensional Scaling. a) mtDNA *F_ST_* and b) Y-STR *R_ST_* distances with c) mtDNA *F_ST_* and d) Y-STR *R_ST_* mean-linkage dendrogram.

HVS-I SNPs were constructed against CRS [62] as revised rCRS [63], and the subrange common to all publications was selected. ARLEQUIN [64] was employed to compute F_ST_'s [65], which were used as distances for non-metric MDS analysis [66], as implemented in isoMDS [61] in R. Agglomerative clustering with mean linkage was applied to the F_ST_ distances in the same way that they were applied to the Euclidean distances as described in the PCA section. An identical MDS and clustering were applied to Slatkin's R_ST_ distances [67] obtained from Y-chromosome samples. These results were displayed in Figures 3.

#### Heatmap

**Figure 4:**
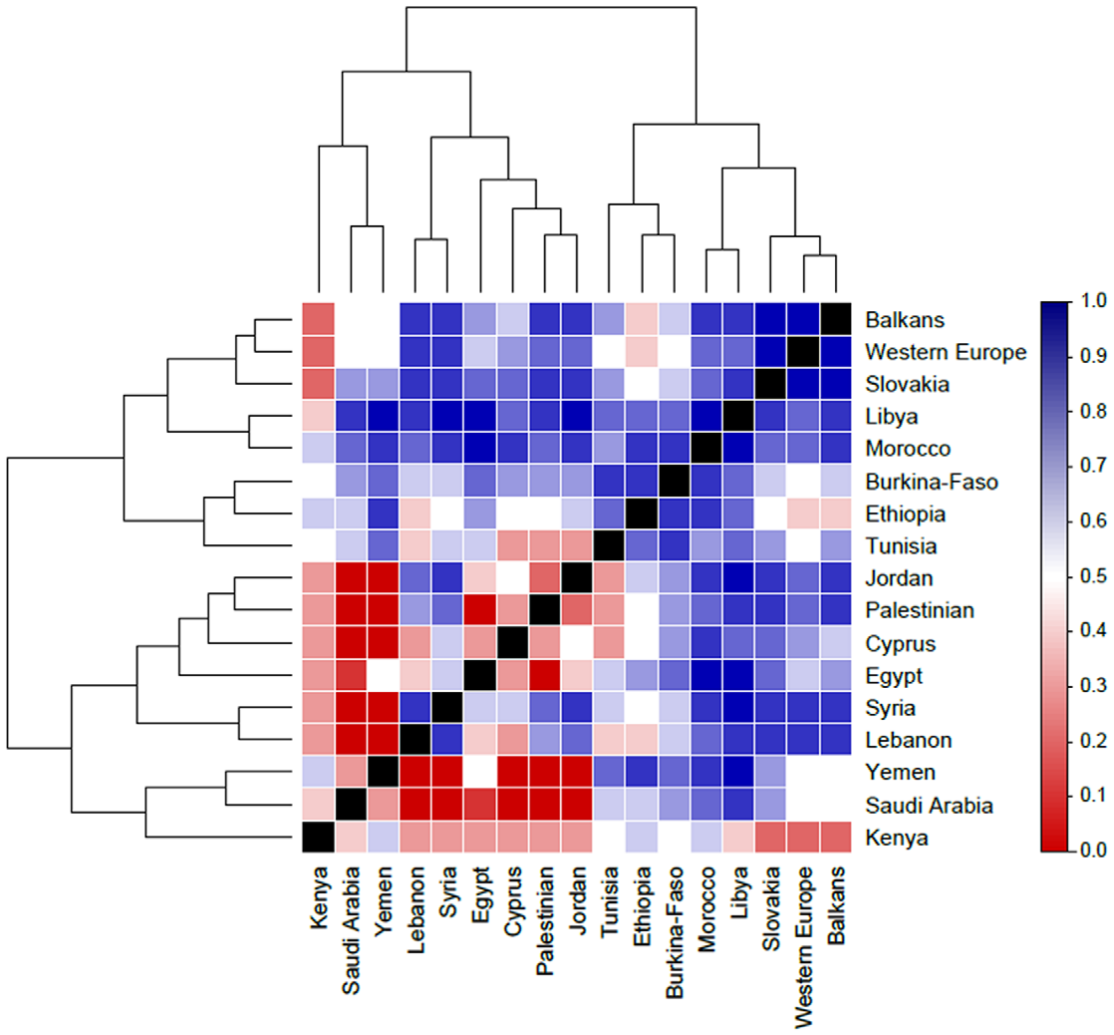
Heatmap of the Y(Y+mtDNA) distances. The heatmap shows the normalized ratio of the Y *F_ST_* distance with respect to the total distance (Y *R_ST_*+mtDNA *F_ST_* distances). The dendrograms are obtained using complete linkage hierarchical clustering with the Euclidean distance measure.

Relative comparisons between mtDNA F_ST_ and Y-chromosome R_ST_ distances were constructed using a heatmap based on the normalized ratio of the Y-chromosome R_ST_ distance with respect to the total distance (R_ST_/(R_ST_+F_ST_)) for mtDNA HVS-1 F_ST_ and Y-chromosome R_ST_ distances (Figure 4). The dendrograms are obtained using complete linkage hierarchical clustering with Euclidean distances between Y/(mtDNA HVS-I+Y) scores.

#### AMOVA

The agglomerative clusters reflecting the results from the MDS analysis were used to identify groups of populations representing affinity of Middle Eastern populations with European and African populations observed in Y-chromosome and mtDNA genetics. AMOVA [68] was applied to the mtDNA and Y sets for each of the mtDNA and Y affinity sets, yielding a 2 by 2 measure of the differences between mtDNA and Y affinities, reported in Table 2.

**Table 2:** mtDNA vs. Y-chromosome AMOVA results contrasting mtDNA and Y dendrogram-based classifications.

		mtDNA dendrogram based affinities				Y-Chromosome dendrogram based affinities			
		Variation	% Variation	F	p-value	Variation	% Variation	F	p-value
mtDNA AMOVA	Within Populations	2.43	97.15	0.029	<10^−4^	2.43	97.59	0.024	<10^−4^
	Within Groups	0.028	1.11	0.014	<10^−4^	0.045	1.84	0.018	<10^−4^
	Between Groups	0.043	1.74	0.017	3.6×10^−3^	0.014	0.57	0.006	1.1×10^−3^
Y-Chromosome AMOVA	Within Populations	6.56	87.05	0.129	<10^−4^	6.03	89.04	0.11	<10^−4^
	Within Groups	0.456	6.05	0.065	<10^−4^	0.329	4.86	0.052	<10^−4^
	Between Groups	0.519	6.89	0.068	6.3×10^−3^	0.414	6.11	0.061	<10^−4^

## Results

### Phylogeographic distribution of mtDNA haplogroups

A total of 185 distinct HVS-I SNPs were identified across all populations (Table S5). The distribution of mtDNA haplogroups shows systematic variation with geography.

The haplogroups' geographical distribution shows affinity between the Northern Levant (modern day Lebanon and Syria) and Europe with clear distinctions between the Levant and the Arabian Peninsula with regards to Africa (Fig. 1, Table 1). The main mtDNA haplogroups for both Europe and the Northern Levant are H and R*. The subhaplogroup H is more frequent in Europe (45%) than in the Levant (25%). Among the Levantine populations, only the Lebanese share Western Europe's overrepresentation of H.

Fisher exact tests were applied to determine when haplogroup frequency differences among populations over both Pan-Mediterranean tests (Table S6), and regional Middle Eastern tests (Table S7), were significant. They reveal patterns of significant over- and under- representation of haplogroups marking regional affinities.

In Lebanese, haplogroups H, HV, T, and K are over-represented, while Syrians are overrepresented in haplogroups T and K. Western Europeans show overrepresentation of haplogroups H, K.

By contrast, haplogroups J, R0, and M are significantly overrepresented in Saudis, and underrepresented in Western Europeans. Haplogroup J was also significantly overrepresented in Iraqis, among Palestinians, and Yemenis.

The African haplogroups L* and L3* are very rare (frequencies less than 1%) and underrepresented in Europe, noting that rarity reduces the power of these tests. In the Levant, Lebanese have the lowest frequency for these haplogroups with generally highly significant underrepresentation, The L haplogroups show rather broad penetration into Yemen, with most being significantly overrepresented, with Yemenis being the only population with an overrepresentation of L6. We have not found haplogroup L6 in our Lebanese (N = 980), Syrian (N = 234), and Jordanian samples (N = 290). Further, they are absent from Abu-Amero et al's samples from Saudi Arabia, as well as other published results included in his study. A lower bound on the relative frequency of L6 in non-Yemeni Middle East of *f*≥8.29·10^−4^ would guarantee that at least one or more would be observed at least 95% of the time out of the 3614 samples collected across the data on which we are reporting. Assuming independent sampling following a binomial test, there is 5% or less chance of seeing zero L6 by chance with a relative frequency of *f* or higher, establishing 8.29·10^−4^ as an upper bound to the relative frequency of L6 with a 95% confidence.

We note that subtypes HV0, HV1 and HV2 are generally too weakly represented among our populations to yield tests with adequate power. HV0 and HV1 show sufficient power when pooled into regions. We note that HV0 appears primarily in Europe (34 out of 42 HV0 samples are European, with p<0.0001), HV1 is primarily non-European (4 out of 60 were European, with 20 among the African sample, and 36 among Middle Eastern samples, p<0.0001). HV2 were very rare, with no significant p-values.

Some U subhaplogroups show regional localization, but none of them rose to sufficient frequency to make any significant contribution to the PCA. U3 appears most frequently in Jordan (Fisher's test: p = 2.39e-8), with representation throughout the Middle East. U4 (p = 1.49e-7) and U5 (p = 2.2e-16) appear to be more heavily European.

The two leading principal components displayed in Figure 2 capture 47.9% and 26.9% of the variance showing a well-defined separation between Mediterranean African populations and sub-Saharan populations (Fig 2a). There is a clear cluster of North African populations comprised of Libyans, Moroccans, and Tunisians. The Nile River marks another boundary of mtDNA differentiation within Africa, linking Egypt, Ethiopia and Kenya but also extending through to Yemen. Yemenis and Saudis both associate strongly with Egyptians, whereas the Jordanian, Lebanese, Palestinian, and Syrian populations clustered together. Thus, the Arabian Peninsula population clusters were relatively differentiated from the more northern Levantine populations.

Mitochondrial DNA Haplogroups showing significant contributions to the principal components include H, L3, L2, L0, V, L1, M, J, U, T, K, HV, and R0. The principal vectors for HV, T, K, J, and U point almost directly at the Levantine cluster (Fig 2a). H marks Western Europe and is a significant contributor to Libyan Sahara and Mali mtDNA diversity. L2 and L3 frequencies distinguish the populations of Kenya, Niger, Burkina Faso, Mali, Tunisia, and Libyan Sahara, with a decrease in frequencies of L haplotypes from Kenya through Saudi Arabia.

The dendrogram based on mtDNA haplogroup frequencies (Fig 2b) reveals the strongest differentiation across the Sahara, showing the northern populations differentiated from the southern ones (with Nigeria, Kenya, Mali, Libyan South Sahara, and Burkina-Faso). Egyptian, Yemeni, Saudi Arabian, and Ethiopian populations form a cluster that is distinct from the rest of North Africa, the remaining parts of the Middle East, and Europe. Among these, Libyans, Moroccans, and Tunisians, form a cluster.

UPGMA and PCA showed Yemenis and Saudis (two of the STR predicted Hg populations) closely associated, forming a clear outlier to clusters identifying more northerly Middle Eastern populations and Europe. Slovaks (the third predicted population) also formed a distinct outlier to all of these. Africans were partitioned into northern African populations and Sahel populations, and distinct from the other populations. Burkinabe formed a very distinct outlier to every other population.

MDS analyses were performed using mitochondrial HVS-I based *F_ST_* (Table S8) and Y-chromosome STR *R_ST_* (Table S9) data (Figure 3a & 3b). The *F_ST_*'s computed with mtDNA HVS-I data and *R_ST_*'s computed from Y-STRs of Ethiopian and Levantine populations tended to be less than 1/3, with *Nm*>1 [69], roughly corresponding to gene flow between populations over the course of time [5], [20], [38], [70], [71].

For the mtDNA HVS-I *F_ST_* MDS analysis, the European populations formed a clear cluster very close to the Cypriots, Jordanians, Lebanese, Palestinians, and Syrians. Egyptian, Libyan, Moroccan, and Tunisian populations form a clear cluster. Significantly, Yemenis are on the far side of North Africans, distinct from the Levantine populations and the Libyan Sahara population stands significantly separated from the North African group. The sub-Saharan populations are clearly distinguished from the Mediterranean populations and show significant distances between them in comparison to the Mediterranean populations. The mtDNA HVS-I MDS and dendrogram show most of the Levantine and Arabian Peninsula populations clustering together. Significantly, Yemenis do not seem to cluster with proximal African populations or with Saudis. The entire Levant population seems to cluster with Western Europeans, Southeastern Europeans, and Slovaks.

In contrast to mtDNA, the Y-STR-based MDS shows a tight cluster of Cypriots, Egyptians, Jordanians, Lebanese, Palestinians, Saudis, Syrians, and Yemenis, though Libyans, Tunisians, and Moroccans extend away from this cluster. The Southeastern Europeans, Slovaks, and Western Europeans lie in the opposite direction. The dendrogram shows a European cluster closer to the Levant/Arabian Peninsula cluster and the North African cluster acting as out-group to those.

In general, the MDS plots for mtDNA and Y-STRs show general agreement of European populations extending from the Levant in one direction and North Africans tending to extend in another direction. This places the Levant as a middle ground, either by averaging of in-migration, as a source feeding both North African populations and European populations, or both. The Y and mtDNA MDS plots differ in identifying affinities of Lebanese with Europeans and Yemenis with Egyptians.

### Comparative analyses of paternal and maternal lineages in the Levant

The relative distance heatmap plot (Figure 4) shows proportion of genetic distances of mtDNA vs. Y. Red colors indicate greater distance of mtDNA vs. Y, while blue colors indicate greater distance of Y vs. mtDNA. Hierarchical clustering organizes the plot relating populations showing similar profiles of Y vs. mtDNA isolation. Most striking is that Saudis, Kenyans, and Yemenis cluster together away from Lebanese, Syrians, Palestinians, Cypriots and Jordanians in terms of showing relatively high differentiation of mtDNA vs. Y-chromosome genetics. Dendrograms provide a consistent description of the organization of data that may be easily compared with PCA or MDS plots. The application of mean-linkage dendrograms to Y STR data, mtDNA HVS-I data, and mtDNA haplogroup frequency data provides a consistent basis of comparison. Application of AMOVA to clustering results provides an independent test characterized by p-values and percent variances between vs. within groups. We are not inferring relationships of heritage among populations by application of mean-linkage clustering.

In order to preserve normalization, common subsets comprised of the 11 populations in common in both dendrograms were included. Each of the candidate partitions marking mtDNA affinities and Y affinities formed three groups. The groups representing mtDNA affinities were: (1) Southeastern Europeans, Lebanese, Slovaks, and Western Europeans vs. (2) Cypriots, Jordanians, Palestinians, Saudis, and Syrians, vs. (3) Egyptians and Yemenis. The groups representing Y affinities were: (1) Southeast Europeans, Slovaks, and Western Europeans vs. (2) Cypriots, Lebanese, Syrians, and Yemenis, vs. (3) Egyptians, Jordanians, Palestinians, and Saudis. These two affinity groupings were applied to both the Y and the mtDNA data, yielding results presented in Table 2. Both Y and mtDNA tend to cluster African, European, and Middle Eastern populations separately, and all combinations showed highly significant between-group vs. within-group variations. This reflects the dominating clustering distinguishing Africa, Europe, and the Middle East populations that mean-linkage clustering is picking up. Affinities of Lebanese and the Levantine populations with Europeans vs. Africans depend on comparisons of AMOVA variations within and between groups. Notably, the mtDNA affinity grouping increased AMOVA between-group variation of mtDNA HVS-I data by a factor of 3.05 compared to the result obtained applying the Y affinity grouping to the mtDNA HVS-I data, and decreased AMOVA within-group variation by a factor of 1.66. However, application of the Y affinity grouping reduced AMOVA between-group variation in Y STR data by a factor of 1.13 while reduced AMOVA within-group variations in the Y STR data by a factor of nearly 1.2 compared to the mtDNA affinity grouping. These factors are relatively neutral in contrasting Lebanese Y-chromosome affinity with Europe vs. North Africa, and actually place Lebanese Y-chromosome organization closer to Europeans than Africans. It is expected that mean-linkage clustering would minimize AMOVA within-groups variation, leading to larger AMOVA between-groups variation. Observation did not meet expectation. Instead, the AMOVA within groups' variations for the Y-chromosomes were reduced using the mtDNA clustering compared to Y clustering, suggesting reduced discrimination using the Y clustering for Y-STR genetics.

### Limitations

Y Chromosome haplogroup frequency analyses are limited by a relatively high misclassification rate, with more than half of the populations showing more than 10% misclassification, and Ethiopia showing nearly 50%. Since PCA is a non-linear computation which folds in all populations, the apparent locations of any two populations may shift relative to each other when a third population is added or distorted.

## Discussion

Here we present mitochondrial characteristics of a large group of newly typed samples from five populations (Lebanese, Libyans, Jordanians, Palestinians, and Syrians) and compare their geographical affinity, distribution, and frequency with those of Y-chromosome markers from populations across the broader region of Africa, Europe and the Arabian Peninsula.

The Y-chromosome results of the current study are in agreement with previous studies, suggesting a Middle Eastern gene pool with greater affinity to Africa. Maternal lineages of the Levantine populations studied here, however, reveal stronger European genetic affinities, while not showing Arabian peninsular influences.

### The contrast between the two lineages

Our results show a contrast of mtDNA affinities with previous Y-DNA results [25]. While our Y-DNA MDS and mean-linkage clustering showed a much greater proportion of East African and Near East Y-chromosomes in the Levant, evidence of much less mtDNA affinity, however, was found between the Levant and its southern neighbours.

European mtDNA affinity with the Levant was established in haplogroup frequency data through Fisher exact tests, PCA, and mean-linkage clustering based on Euclidean distances, and in HVS-I derived *F_ST_* distances via MDS and mean-linkage cluster analysis. The mtDNA results are distinct from the Y-STR *R_ST_-*based mean-linkage cluster analysis that showed closer affinity of the Levant populations with Cypriots, North Africans, and Yemenis, than to Europeans.

This cluster analysis suggests that the position of Lebanese relative to European Y-chromosome genetics represented in STR haplotype data is also much more ambiguous than suggested entirely by frequency analysis, revealing otherwise cryptic relationships between Lebanese's Y-STR structure and that of Europeans. Cluster analysis of Y-chromosome frequency based data shows similar partitioning of Europe, Africa, and Middle East, with the Levant much more strongly associated with the Middle East than Europe. As with mtDNA, African Y-chromosome haplogroup data also shows a clear partition between Northern populations and Sahel populations. Due to uncertainties in haplogroup inference from STRs, affinities of Yemenis with Ethiopians vs. Egyptians are uncertain, as are the relationships of Saudi Arabian haplogroups both similar to Yemenis or differentiated from Yemenis in affinity with African populations.

### The Levant and Europe

Beyond the associations noted above, Lebanese show affinity with Europeans for mtDNA haplogroups H, HV, T, K, J, and U, all of which have been identified as markers of agricultural expansions from the Fertile Crescent into Europe [17].

Colonization of West Eurasia by modern humans is believed to have been a consequence of the Out-of-Africa dispersal and to have occurred via the Levant [6]. Indeed, migrating modern humans are believed to have settled near the Arabian Sea until climate changes allowed them to reach the Levant and then Europe [17], [72]–[74]. The LGM, followed by re-expansions from smaller LGM communities relatively isolated by widespread arid conditions, further impacted the coastal-inland contrast of Y-chromosome genetics [25], [75]. The significant overrepresentation of mtDNA haplogroup HV among Levantine populations compared to their southern neighbours has suggested these lineages were most likely derived from a single maternal Levantine source population [17].

### Arabian genetic expansions: Arabia East Africa, and North Africa

From the 7^th^ millennium B.C.E., empire expansions and trade, including the slave trade, heavily influenced genetic migration between Yemen and East Africa. Alternatively, known trade networks linking Egypt with Yemen included those for obsidian, and later through Aksum, spices, incense and other precious materials, as well as slaves [76]–[78]. It is particularly clear from prior mtDNA studies of this region that East African migration into Arab populations involved females to an extensive degree [79]. While Ethiopian and other East African populations may appear to be better candidates for the origins of modern Yemeni populations, our PCA and MDS analyses, and their associated mean-linkage clustering of Yemen's mtDNA, show greater affinity between Yemenis, Egyptians and North Africans. They share in common haplogroups J, L0, L2, and N1. Comparison of mtDNA HVS-I *F_ST_* distances also suggest that Yemen appears more similar to Egypt than Ethiopia.

Two haplogroups in this region show significant evidence of relative isolation. First, mtDNA patterns for haplogroup J reflect relatively moderate genetic outflow from Saudi Arabia, and haplogroup L6 is strongly localized within Yemen. Haplogroup J is evenly distributed throughout the Middle East, except in Saudi Arabia where it is significantly overrepresented.

It is likely the pattern of Hg J's significant penetration, and the shared underrepresentation of Hg H, tips the balance for Yemenis' mtDNA affinity with Egyptians. Given the significant underrepresentation of Hg J in East Africa, while not being significantly uncommon in Egypt, it is therefore plausible that Arabian female gene flow followed well established trade routes on the Red Sea with Egypt and North Africa while avoiding assimilation of Yemeni L6's on the way.

The most striking feature of the heat map (Fig 4) is the relative isolation of mtDNA genetics of Yemenis and Saudis from the other populations in the Middle East in comparison to Y-chromosome variation. While Yemenis appear to share overrepresented haplogroups that characterize each of its neighbouring populations, none of the African populations have become dominated by Saudi Arabian J's, nor have Middle Eastern populations been differentially dominated by the in-migration of African L's the way Yemenis have.

The expansion of trade through the Red Sea and into the Indian Ocean basin starting in Classical times has provided the largest opportunities for genetic transfers from Africa into Yemen, being dominated by the Red Sea superpower: Egypt. The distribution of mtDNA haplogroup L6 provides a measure of the limited impact of genetic outflow from Yemen, and this flow seems to have been primarily unidirectional. This establishes the upper limits for Yemeni female-mediated gene flow during the Muslim Expansions, as well as identifying possible routes for the expansions.

Whether considering haplogroup composition revealed in Fisher tests, PCA, or *F_ST_* based MDS analysis of HVS-I data, mtDNA shows a much stronger affinity between Levantine populations and Europeans compared with the rest of the Middle Eastern populations, or with North Africans. While Lebanese and Yemeni mtDNA epitomize very distinct affinities to different populations and regions well outside of the Middle East, Saudi Arabia seems to display strong local over-representation haplogroup J, while Yemen is even more localized in its L6. Further, these large-scale differences in affinity between mtDNA genetics appear in sharp contrast to regional affinities seen in their Y-chromosomal counterparts. While the mtDNA signal is sharp and clear in its affinities, the Y-chromosome results show somewhat more ambiguous associations in *R_ST_* based analyses, with Lebanese showing less within-group variation when organized consistently with mtDNA and demonstrating associations closer to Europeans than Africans. This would suggest that while male migrants accompanied female migrants, especially to Europe, females did not always accompany male migrants, especially into North Africa. This leaves a more ambiguous signal for male compared to female migrations.

The historical and archaeological record reveals how trade and labour, colonization and settlement events, and military expansions all contributed to the immigration and displacement of individuals throughout these regions. As a distinct crossroad between geographic regions and civilizations, the Levant and the Near East harbour unique genetic affinities which are revealed most clearly through the comparison of Y-chromosome and mtDNA data.

Due to uncertainties in haplogroup inference from STRs, specific questions regarding affinities of Yemen with Ethiopia vs. Egypt are inaccessible, as are questions regarding the relationship of Saudi Arabian haplogroups both similar to Yemenis or differentiated from Yemenis in affinity with African populations.

## Supporting Information

Figure S1 **Geographic distribution of Y haplogroups.** Frequencies from published data as reported in Table S3.
(TIF)

Figure S2 **Populations comparison based on Y haplogroups** a) Principal Component Analysis of relative frequencies of Y haplogroups within populations, b) with mean-linkage (UPGMA) dendrogram determined from Euclidean distances.
(TIF)

Table S1 **mtDNA haplotypes analyzed in this study.**
(XLS)

Table S2 **Y chromosome STR haplotypes and haplogroups employed in this study.**
(XLS)

Table S3 **Y chromosome Haplogroup frequencies of populations used in this study.**
(XLS)

Table S4 **List of the mtDNA Haplogroup marker SNPs typed for this study.**
(XLS)

Table S5 **List of HVS-I mtDNA SNPs identified across populations used in this study.**
(XLS)

Table S6 **Fisher exact tests for haplogroup frequencies vs. population across all study populations.**
(XLS)

Table S7 **Fisher exact tests for haplogroup frequencies vs. population within the Middle East.**
(XLS)

Table S8 **mtDNA** ***F_ST_*** **distances between populations.**
(XLS)

Table S9 **Y STR** ***R_ST_*** **distances between populations.**
(XLS)
