# Supplementary figures and images for: Y-Chromosome and mtDNA Genetics Reveal Significant Contrasts in Affinities of Modern Middle Eastern Populations with European and African Populations
Source: PLoS One. 2013 Jan 30;8(1):e54616. doi: 10.1371/journal.pone.0054616 (PMC3559847; doi:10.1371/journal.pone.0054616)

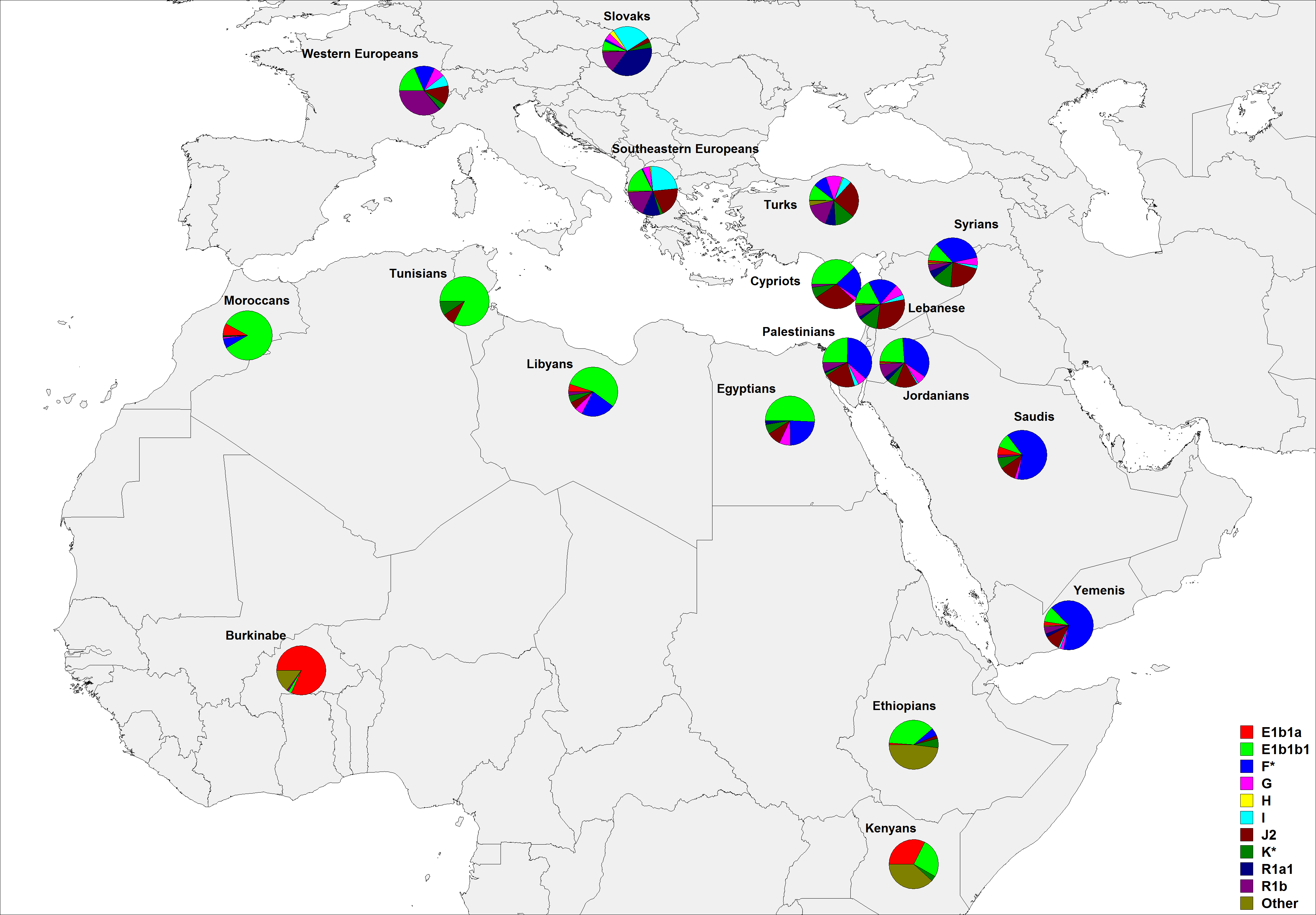

Supplement: Figure S1 — Geographic distribution of Y haplogroups. Frequencies from published data as reported in Table S3. (TIF) [file pone.0054616.s001.tif]

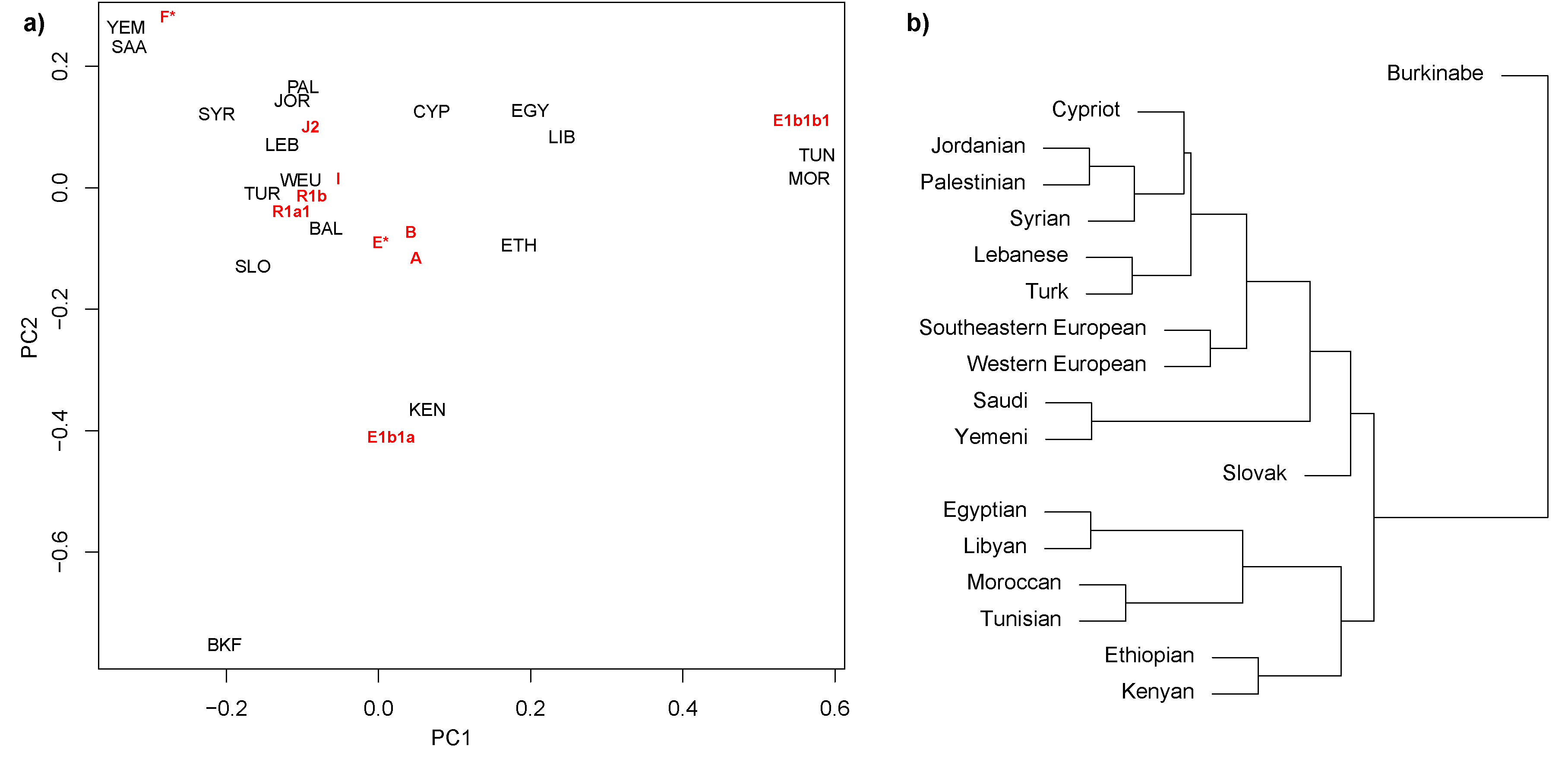

Supplement: Figure S2 — Populations comparison based on Y haplogroups a) Principal Component Analysis of relative frequencies of Y haplogroups within populations, b) with mean-linkage (UPGMA) dendrogram determined from Euclidean distances. (TIF) [file pone.0054616.s002.tif]
